# Fully Automated AI-Based Digital Workflow for Mirroring of Healthy and Defective Craniofacial Models

**DOI:** 10.3390/jimaging11110407

**Published:** 2025-11-12

**Authors:** Michel Beyer, Julian Grossi, Alexandru Burde, Sead Abazi, Lukas Seifert, Joachim Polligkeit, Neha Umakant Chodankar, Florian M. Thieringer

**Affiliations:** 1 Department of Oral and Cranio-Maxillofacial Surgery, University Hospital Basel, 4031 Basel, Switzerland; 2 Medical Additive Manufacturing Research Group (Swiss MAM), Department of Biomedical Engineering, University of Basel, 4123 Allschwil, Switzerland; 3 Department of Prosthetic Dentistry and Dental Materials, Iuliu Hatieganu University of Medicine and Pharmacy, 32 Clinicilor Street, 400006 Cluj-Napoca, Romania; 4 Department of Dentistry, Healthway Hospitals, Ella Old Goa 403110, Goa, India; 5 Miramar Dental Centre, Caranzalem, Panaji 403001, Goa, India

**Keywords:** artificial intelligence, image processing, computer simulation, virtual surgical planning

## Abstract

The accurate reconstruction of craniofacial defects requires the precise segmentation and mirroring of healthy anatomy. Conventional workflows rely on manual interaction, making them time-consuming and subject to operator variability. This study developed and validated a fully automated digital pipeline that integrates deep learning–based segmentation with algorithmic mirroring for craniofacial reconstruction. A total of 388 cranial CT scans were used to train a three-dimensional nnU-Net model for skull and mandible segmentation. A Principal Component Analysis–Iterative Closest Point (PCA–ICP) algorithm was then applied to compute the sagittal symmetry plane and perform mirroring. Automated results were compared with expert-generated segmentations and manually defined symmetry planes using Dice Similarity Coefficient (DSC), Mean Surface Distance (MSD), Hausdorff Distance (HD), and angular deviation. The nnU-Net achieved high segmentation accuracy for both the mandible (mean DSC 0.956) and the skull (mean DSC 0.965). Mirroring results showed minimal angular deviation from expert reference planes (mandible: 1.32° ± 0.71° in defect cases, 1.58° ± 1.12° in intact cases; skull: 1.75° ± 0.84° in defect cases, 1.15° ± 0.81° in intact cases). The presence of defects did not significantly affect accuracy. This automated workflow demonstrated robust performance and clinical applicability, offering standardized, reproducible, and time-efficient planning for craniofacial reconstruction.

## 1. Introduction

Craniofacial reconstruction is required in a range of clinical situations, from trauma and tumor resections to congenital deformities and infections. Damage to the mandible is particularly frequent in oncologic head and neck surgery, especially after segmental resections for oral squamous cell carcinoma, but also occurs after fractures [1]. Skull defects, on the other hand, often follow decompressive craniectomy, tumor removal, or craniosynostosis correction. Both structures are essential for facial harmony, structural stability, and functional integrity [2,3]. Loss of continuity in the mandible can severely limit mastication, speech, and appearance, while cranial defects risk compromising brain protection and may produce neurological or cosmetic consequences.

Advances in surgical planning have transformed how these reconstructions are performed. Virtual Surgical Planning (VSP) and Computer-Aided Design/Computer-Aided Manufacturing (CAD/CAM) systems now allow detailed visualization of the patient’s anatomy in three dimensions, the simulation of surgical steps, and the creation of customized cutting guides, repositioning templates, or patient-specific implants (PSIs). Since the earliest three-dimensional reconstructions from CT data in the 1980s [4,5,6] and the introduction of CAD/CAM into dentistry in 1989 [7], these technologies have evolved into fully digital workflows [8] that no longer require physical models [9]. Today, the integration of radiologic imaging with computer-based planning and fabrication methods has been shown to improve surgical precision, shorten operating time, and enhance both functional and cosmetic outcomes.

Mirroring is one of the most valuable digital techniques in craniofacial surgery. For the mandible, it can be used to quantify a defect, guide prebending of plates, or reconstruct fractures by reflecting the healthy side onto the affected one. This allows PSIs or plates to be shaped for an exact fit, minimizing adjustments during surgery and improving reconstruction accuracy [10,11]. In the skull, mirroring is essential for reconstructing fracture patterns, repairing orbital walls, or producing implants after craniectomy [12,13]. The mirrored geometry can serve as the basis for manufacturing PSIs in materials such as PMMA or PEEK, ensuring that implants match the patient’s anatomy [14].

### Related Work

While planning technologies have advanced significantly, creating accurate segmentations of the skull and mandible remains a critical and often difficult step. The skull’s extensive and complex surface, together with the mandible’s intricate geometry, make manual segmentation both time-consuming and operator-dependent. Artificial intelligence–driven methods, particularly deep learning models like nnU-Net, have recently demonstrated strong potential in this field by providing fast, accurate, and reproducible segmentations [15]. The nnU-Net framework includes an automatic adaptation of its architectures to a given image geometry [16].

Different computational approaches exist for generating mirrored reconstructions. Landmark-based techniques, used initially in facial asymmetry studies [17,18], define the symmetry plane by connecting or averaging bilateral landmarks, but are prone to user variability. Algorithmic methods use segmented anatomy to mathematically calculate symmetry, often relative to the mid-sagittal plane. Examples include the PCA–ICP method described by Noori et al. [19], which estimates a symmetry plane using principal component analysis followed by iterative closest point alignment, and the ICP-based surface optimization method from De Momi et al. [20]. Other 3D techniques automatically calculate the craniofacial symmetry midplane from CT scans using convolutional neural network (CNN) and geometric moments [21]. Although efficient and fully automated, these methods work best when anatomical symmetry is relatively preserved. Deformable image registration can overcome asymmetry by warping the anatomy to align both sides, but at the cost of high computational demand and a requirement for high-resolution imaging. More recently, deep learning has been applied to symmetry detection, as in PRS-Net, which can locate symmetry planes even in noisy or incomplete datasets [22], and in the mandibular midsagittal plane estimation method described by Wang et al. [23].

Studies on the comparison of mirroring with anatomy-based curve reconstruction suggest the use of either of the techniques based on the size of the defect. Mirroring was found to be advantageous in medium to larger defects [24]. While prior studies focus on symmetry plane detection or segmentation, the present study introduces an integrated and fully automated pipeline that combines AI-based segmentation of the skull and mandible with an automated mirroring process with defect modeling. The system reflects the unaffected orbit or mandibular side onto the defect area, producing a reconstruction that is both anatomically accurate and clinically applicable. This combination is intended to improve precision, efficiency, and usability in reconstructive surgery, supporting functional, aesthetic, and urgent interventions, and to evaluate the performance of this workflow in both healthy and pathological anatomy.

## 2. Materials and Methods

This section outlines the methodology used to develop and train the artificial intelligence system for cranium and mandible segmentation, as well as the mirroring algorithm. It includes descriptions of data collection and preprocessing, image segmentation, AI model training, postprocessing procedures for result refinement, and performance evaluation through validation metrics and statistical analysis.

### 2.1. Data Acquisition

For system development, 388 head and neck CT scans were obtained from the University Hospital Basel. The dataset included a wide range of imaging modalities and acquisition protocols to maximize variability. The inclusion strategy was designed to create a segmentation system that performs reliably across diverse clinical scenarios.

On average, the scans contained 331.56 ± 207.42 slices, with mean voxel dimensions of 0.77 ± 0.61 mm in the X direction, 0.91 ± 0.77 mm in the Y direction, and 1.18 ± 0.91 mm in the Z direction. To ensure uniform resolution, all images were resampled to isotropic 0.5 mm voxels using a B-spline interpolation algorithm. The processed datasets were stored in Digital Imaging and Communications in Medicine (DICOM) format. The data were split into a training set of 348 scans and a test set of 40 scans. The test set consisted of 20 scans containing the mandible (10 with defects such as atrophy of the mandibular body, condylar atrophy, or fractures and 10 with intact anatomy) and 20 scans containing the complete skull (10 with defects including craniotomies, patient-specific implants, or fractures and 10 with intact anatomy).

Ethical approval was granted by the Ethics Committee of Northwestern and Central Switzerland (EKNZ BASEC 2023 00446). The study, entitled CT and MRI Segmentation for Detection and Classification of Diseases or Healthy Aging in Radiology, was authorized under the provisions for reuse of health-related personal data for research in accordance with Article 34 of the Swiss Human Research Act (HFG). All datasets were anonymized prior to analysis to comply with data protection regulations. A summary of the main dataset characteristics is presented in Table 1.

### 2.2. Automatic Workflow

#### 2.2.1. Segmentation

The dataset of 348 head CT scans was employed to train a full-resolution three-dimensional nnU-Net model for segmentation of the skull and mandible, as described by Isensee et al. [16]. The underlying network followed a U-Net configuration, using instance normalization, leaky ReLU activation, and a combined Dice cross entropy loss function. The nnU-Net framework automatically selected the preprocessing pipeline, network structure, training schedule, and postprocessing strategy according to the characteristics of the dataset. Parameters including patch size, network depth, and the number of feature channels were adjusted to match the image resolution and the capacity of the available GPU.

Model training was performed on a high-performance workstation equipped with an Intel Core i9 14900 central processing unit (Intel, Santa Clara, CA, USA), an NVIDIA GeForce RTX 4070 Ti SUPER graphics processing unit (NVIDIA, Santa Clara, CA, USA), and 128 GB of DDR6 memory. The process required approximately 20 h to complete.

#### 2.2.2. Mirroring

This automated workflow was developed to identify the sagittal midplane and perform mirroring of craniofacial structures, specifically the mandible and skull, in CT imaging data. The process begins with a binary segmentation of osseous anatomy, obtained by applying a threshold of 250 Hounsfield units (HU) to the CT volume. This segmentation is converted into a three-dimensional point cloud that captures both the geometry and radiodensity values of the targeted region. To standardize spatial resolution and improve computational efficiency, the original CT data are first resampled to an isotropic voxel size of 2.0 mm.

The estimation of the symmetry plane is initiated by applying principal component analysis (PCA) to the point cloud. The principal axis of variation, derived from the largest eigenvector, is used to define the initial plane normal. This preliminary plane divides the dataset into left and right halves. The points from one side are reflected across the plane and aligned to the opposite side using an Iterative Closest Point (ICP) registration. Unlike conventional geometric ICP, this implementation incorporates voxel intensity values alongside spatial coordinates, thereby integrating both morphological and radiodensity information into the alignment process.

During registration, point correspondences exceeding a set maximum distance are excluded to mitigate the influence of asymmetry, bone loss, or image noise. The rotation resulting from ICP is expressed in axis–angle form and applied to adjust the plane normal. The process is repeated for several iterations, typically between five and ten, until the plane orientation converges to a stable position that best represents the anatomical midline. This iterative refinement concept aligns with methodological principles described by Noori et al. [19].

### 2.3. Manual Workflow

#### 2.3.1. Segmentation

The finalized segmentations of the mandible and bony skull were exported in Standard Tessellation Language (STL) format for downstream analysis. These were generated in the Mimics Innovation Suite (Version 25.0, Materialise NV, Leuven, Belgium) by clinical experts following a semi-automated, standardized protocol. The process made use of tools such as Threshold, Split Mask, Region Grow, Edit Mask, Multiple Slice Edit, Smart Fill, and Smooth Mask. Attention was given to precise delineation of complex anatomical regions, including the condyles, dentition of both jaws, and thin cranial structures such as the orbital floor and the lateral wall of the maxillary sinus.

#### 2.3.2. Mirroring

In the manual mirroring workflow, a symmetry plane was first established to guide reconstruction. For healthy cases, this was achieved by mirroring the right side of the anatomy onto the left, whereas in defect cases the intact side was mirrored onto the side with the defect. The mirrored model was then iteratively aligned with its counterpart to maximize surface congruence, paying particular attention to the correspondence of anatomical landmarks. This alignment process was carried out by a clinical expert, ensuring that the mirrored and original meshes overlapped as precisely as possible. Once optimal alignment was reached, the final symmetry plane was defined to represent the anatomical midline, enabling accurate transfer of geometry from the intact to the affected side. This approach provided a high-fidelity anatomical reference for subsequent evaluation and planning.

### 2.4. Assessments

#### 2.4.1. Segmentation Assessment

Segmentation accuracy of the AI-generated results was assessed in comparison with manual segmentations using both surface-based and volume-based metrics: Dice Similarity Coefficient (DSC), Mean Surface Distance (MSD), and Hausdorff Distance (HD). The definitions and mathematical formulations of these metrics are presented in Table 2. All statistical computations were performed in Python (version 3.9.0).

#### 2.4.2. Mirror Plane Assessment

The agreement between the algorithm-based and manual mirroring approaches was evaluated by comparing the orientation of the symmetry planes obtained from each method. Rotational deviation was determined by measuring the angle between the normal vectors of the two planes, yielding a quantitative assessment of their alignment. All angles were reported in degrees. Statistical analyses were conducted using Python (version 3.9.0). A *p*-value < 0.05 was considered statistically significant (α = 0.05).

## 3. Results

### 3.1. Segmentation Assessment

**Figure 1:**
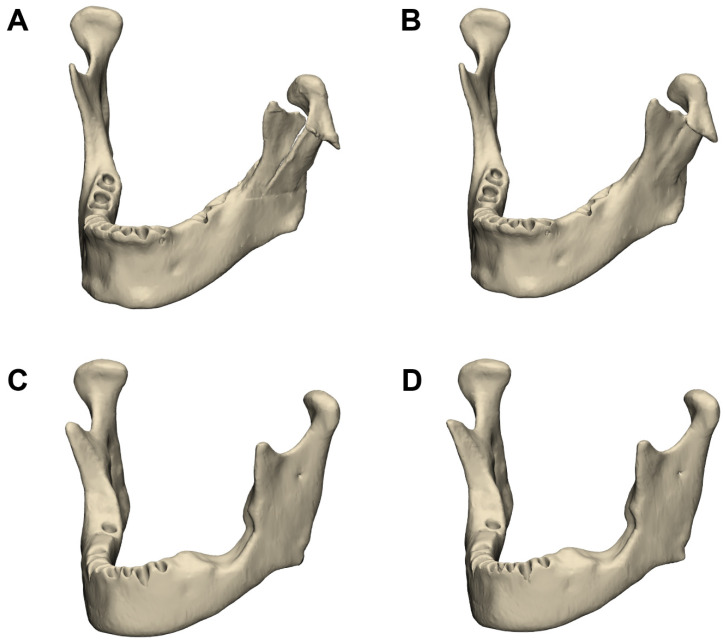
Three-dimensional reconstructions of the mandible segmented from computed tomography data. Panels (**A**,**B**) show a mandible with a fracture on the left side, segmented manually (**A**) and using the AI-based model (**B**). Panels (**C**,**D**) show a mandible with left-sided atrophy, segmented manually (**C**) and using the AI-based model (**D**).

Mandible segmentation using the AI-based method was evaluated on 20 CT datasets, comprising 10 healthy cases and 10 with mandibular defects. The mean Dice Similarity Coefficient (DSC) was 0.956 ± 0.018 (95% CI: 0.95–0.96), with slightly lower values for defect cases (0.952 ± 0.016) compared to intact cases (0.960 ± 0.019). The mean Hausdorff Distance (HD) was 4.47 ± 1.50 mm (95% CI: 3.82–5.13 mm), and the Mean Surface Distance (MSD) was 0.166 ± 0.044 mm (95% CI: 0.15–0.19 mm). Figure 1 shows three-dimensional reconstructions of the mandible in one case with a fracture and another with atrophy, each segmented manually and using the AI-based model.

**Figure 2:**
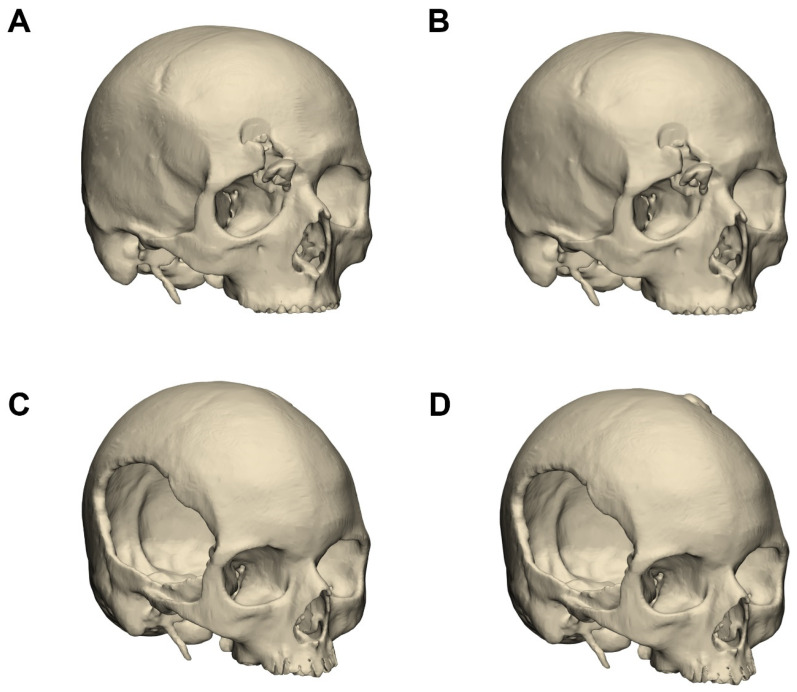
Three-dimensional reconstructions of skulls segmented from computed tomography data. Panels (**A**,**B**) depict a case with a right-sided frontal fracture, segmented manually (**A**) and with the AI-based model (**B**). Panels (**C**,**D**) depict a case with a right-sided cranial defect, segmented manually (**C**) and with the AI-based model (**D**).

**Figure 3:**
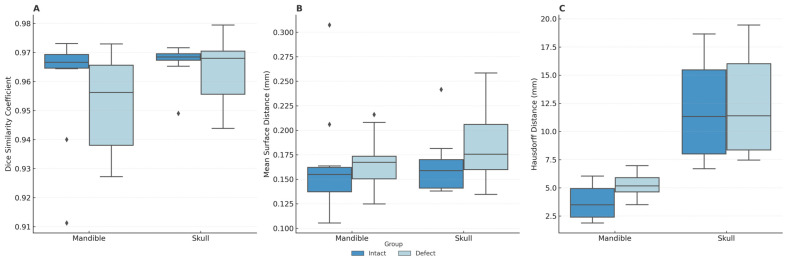
Segmentation accuracy for mandible and skull datasets, stratified by presence or absence of defects. The boxplots illustrate (**A**) Dice Similarity Coefficient, (**B**) Mean Surface Distance in millimeters, and (**C**) Hausdorff Distance in millimeters, comparing results from the AI-based segmentation model with manual reference segmentations.

Skull segmentation performance was evaluated on an additional 20 CT datasets, equally divided between defect and intact cases. The overall mean DSC was 0.965 ± 0.009 (95% CI: 0.96–0.97), with slightly reduced performance in defect cases (0.964 ± 0.011) relative to intact cases (0.967 ± 0.007). The mean HD was 12.16 ± 4.31 mm (95% CI: 10.27–14.04 mm), and the mean MSD was 0.174 ± 0.036 mm (95% CI: 0.16–0.19 mm). Normality of data distribution was assessed using the Shapiro–Wilk test. Results indicated that HD values were approximately normally distributed in both datasets (*p* > 0.05), while DSC and MDS deviated from normality (*p* < 0.05). Based on these findings, non-parametric testing was applied for group comparisons. Specifically, Mann–Whitney U tests were used to compare defect and intact groups within each anatomical category. Only the Hausdorff Distance in the mandible dataset showed a statistically significant difference, with higher values in the defect group (*p* = 0.049). All other comparisons, including DSC and MDS in the mandible as well as all metrics in the skull dataset, did not reach statistical significance (*p* > 0.05). Figure 2 illustrates three-dimensional reconstructions of the skull in one case with a frontal fracture and another with a defect, each segmented manually and using the AI-based model, and the corresponding quantitative comparisons are summarized in Figure 3.

### 3.2. Mirror Plane Assessment

**Figure 4:**
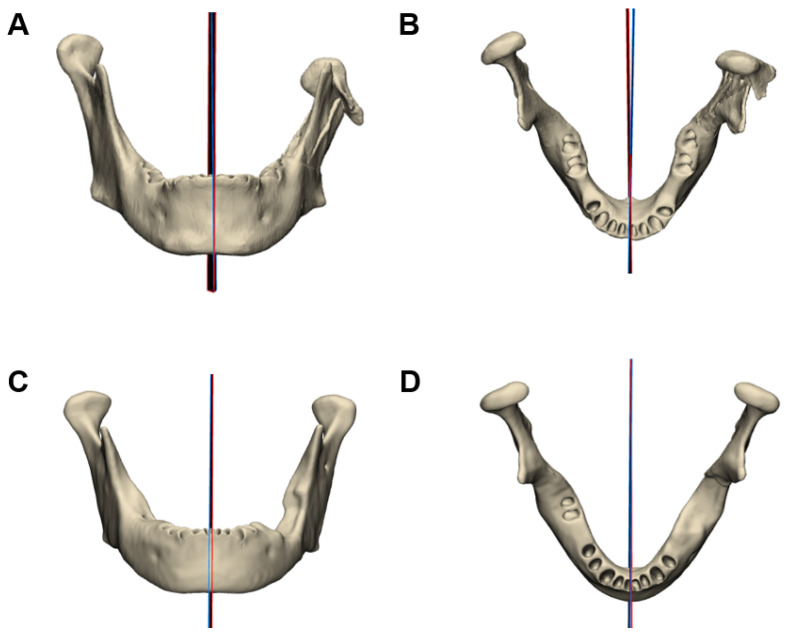
Comparison of automated and manually defined symmetry planes for the mandible. The algorithmically generated plane is shown in blue, and the manually placed plane in red. (**A**,**B**) Mandibular case corresponding to Figure 1A,B, shown from the front (**A**) and top view (**B**). The angular deviation between planes in this case was 1.84°. (**C**,**D**) Mandibular case corresponding to Figure 1C,D, shown from the front (**C**) and top view (**D**). The angular deviation between planes in this case was 0.64°.

**Figure 5:**
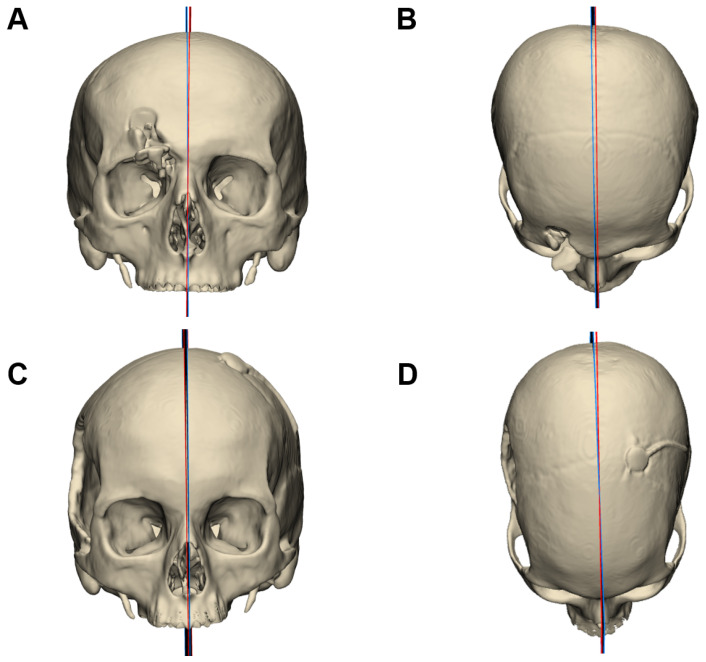
Comparison of automated and manually defined symmetry planes for the skull. The algorithmically generated plane is shown in blue, and the manually placed plane in red. (**A**,**B**) Skull case corresponding to Figure 2A,B, shown from the front (**A**) and top view (**B**). The angular deviation between planes in this case was 0.95°. (**C**,**D**) Mandibular case corresponding to Figure 2C,D, shown from the front (**C**) and top view (**D**). The angular deviation between planes in this case was 1.55°.

**Figure 6:**
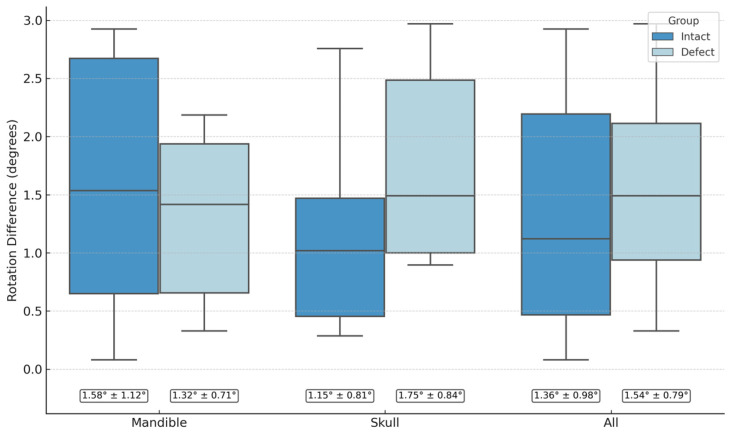
Rotational deviations between algorithmic and manual mirroring for mandible and skull datasets, stratified by defect status.

The evaluation of algorithmic mirroring against expert-defined reference planes was conducted on 40 CT datasets, comprising 20 mandibular and 20 cranial cases, each evenly split into defect and intact groups. Rotational deviation between the normals of the automatic and manual symmetry planes was used as the primary quantitative metric. For mandibular cases, the mean deviation was 1.32° ± 0.71° in the defect group (95% CI: 0.88–1.76°) and 1.58° ± 1.12° in the intact group (95% CI: 0.89–2.27°). In the skull datasets, deviations averaged 1.75° ± 0.84° for defect cases (95% CI: 1.24–2.27°) and 1.15° ± 0.81° for intact cases (95% CI: 0.65–1.65°). The angular deviations were first tested for normality using the Shapiro–Wilk test, indicating no significant deviation from a normal distribution (*p* > 0.05). This justified the use of parametric testing. Independent two-sample t-tests were then performed to compare defect and intact groups within the mandible and skull datasets, revealing no significant differences between defect and intact cohorts within either anatomical category (*p* > 0.05). When both structures were analyzed together, the overall angular discrepancy between automated and manual planes remained consistently low, demonstrating that the presence of cranial or mandibular defects did not adversely impact the accuracy of the mirroring workflow. Figure 4 and Figure 5. Representative cases illustrating the comparison between the automated symmetry plane (blue) and the manually placed reference plane (red) from frontal and axial views. Figure 4 shows mandibular cases, while Figure 5 depicts cranial cases. The distribution of angle deviation results is visualized in Figure 6.

## 4. Discussion

### 4.1. Performance of the Mirroring Algorithm

The automated mirroring workflow produced consistently low angular deviations when compared with expert-defined reference planes. Across all 40 cases, the mean differences remained within a narrow range of approximately one to two degrees. This confirms that the algorithm is capable of generating clinically reliable symmetry planes, independent of whether the anatomy was intact or contained a defect. Importantly, no statistically significant differences were detected between defect and intact groups within either the mandible (1.32° ± 0.71° vs. 1.58° ± 1.12°, *p* = 0.54) or the skull (1.75° ± 0.84° vs. 1.15° ± 0.81°, *p* = 0.12).

### 4.2. Quantitative Benchmarking

The mean angular deviation achieved by the proposed fully automated PCA–ICP workflow (mandible: 1.32° ± 0.71°; skull: 1.75° ± 0.84°) demonstrates performance comparable to, and in some cases superior to, previously reported mirroring algorithms in the cranio-maxillofacial literature. Noori et al. (2020) developed a PCA-ICP–based method for mid-plane detection in intact and unilateral midface fracture skulls and reported Hausdorff distance reductions of approximately 65–66%, corresponding to an equivalent angular deviation of 1.5–2.5° after six iterations [19]. Likewise, Di Angelo et al. (2019) proposed a weighted ICP algorithm coupled with a particle swarm optimization strategy for both unilateral and bilateral cranial defects, achieving mean asymmetry values of 0.66–1.30 mm, corresponding to angular deviations of approximately 1.5–2.0° [24]. When compared to these established approaches, the present workflow shows slightly lower mean angular error across both mandibular and cranial reconstructions, suggesting that the inclusion of radiodensity-weighted refinement and AI-based segmentation enhances the geometric stability and accuracy of the mirrored models. These findings support the clinical reliability of the proposed system and its readiness for translation into automated surgical planning pipelines.

### 4.3. Subgroup-Specific Performance

Interestingly, the mandibular datasets showed slightly better agreement in defect cases compared to intact cases. One possible explanation is that localized mandibular defects leave a clearly defined intact side, which provides both the algorithm and the expert with a stable reference for mirroring. In contrast, complete mandibles frequently exhibit subtle natural asymmetries in condylar orientation, dental alignment, or mandibular body contours. These bilateral variations, although clinically insignificant, can complicate the definition of an exact mid-sagittal plane, which may account for the slightly higher angular deviation observed in the intact group.

For the skull datasets, the opposite trend was observed, with larger deviations in defect cases. Skull defects in this study were typically more extensive and irregular than mandibular ones, including cases of craniectomy or complex cranial fractures. Such large discontinuities disrupt the global geometry, making it difficult for the PCA–ICP-based algorithm to converge on a stable symmetry plane. Furthermore, the absence of large bilateral regions reduces the availability of matching anatomical landmarks, creating challenges not only for the algorithm but also for manual definition by experts. This likely explains the higher deviation in skull defect cases (1.75° ± 0.84°) compared with intact skulls (1.15° ± 0.81°).

### 4.4. Comparison with Literature and Conventional Methods

A fair comparison between the findings of this study and those of other studies was not possible, owing to the limited research available on AI-based digital workflow for the mirroring of mandibular and calvarial models. The study on comparison of mirroring with anatomy -based curve reconstruction reported favorable results in smaller mandibular defects of less than 20 mm with anatomy curve method while mirroring was found to be superior in case of medium and large mandibular defects. Although smaller resections may benefit from anatomy guided interpolation, the present fully automated pipeline is superior and more advantageous in moderate to extensive defects [25].

A whole surface analysis study by Gibelli et al. on zygomatic bones in unaffected subjects reported a mean point to point distance near zero and Root Mean Square (RMS) distances of about 0.84 mm, thus establishing the physiological limits of asymmetry [26]. This substantiates that the observed 1–2-degree plane deviation and sub-millimetric to millimetric surface distances within these set baselines are clinically acceptable. This suggests that the small angular and surface deviations observed in the present analysis fall within the expected normal variation.

The complexity of the condyle ramus unit may greatly influence the outcome of these techniques. A reduced segmental mirroring accuracy was noted in an analysis conducted by Davies et al. on simulated mandibular reconstructions in pathologies involving the condyle [27]. A quantitative conformance testing across Brown defect types demonstrated sub-millimetric RMS conformance while a definite reduction in the accuracy was noted in condyles and to a lesser extent in coronoids. This observation is in congruence with the present study behavior of symmetry-plane estimation in anatomically complex regions. Hence, the use of selective adjuncts is indicated when temporomandibular joint structures are involved. Independent 3D assessments in adults without any overt asymmetry showed the highest level of asymmetry at the condyle, followed by the ramus and corpus with a subtle side predilection. The regional variability in the present pipeline may also be partially attributed to these baseline patterns noted by España-Pamplona et al. [28]. This justifies a stratified accuracy reporting based on subregions.

Robust, automated, landmark-independent mid-sagittal plane algorithms proposed by Di Angelo et al., outperformed the conventional PCA or ICP alone even in case of large defects and gross asymmetries [24]. In contrast the use of PCA–ICP approach in the present study performed reliably in defective anatomies. Future enhancements should aim at incorporation of robust objective terms or outlier mitigation before ICP in cases with severe asymmetry.

A standardized postoperative analysis framework by El-Mahallawy et al. has demonstrated excellent agreement between virtual measurements and the actual postoperative outcome in virtually assisted mandibular reconstructions [29]. This high reliability between the planned and achieved reconstructions indicates that geometric endpoints can be transferred to clinical outcome assessment. Thus, incorporation of such protocols will enable validation of symmetry-plane accuracy against functional and occlusal metrics. 

The convergence of evidence indicates that automated mirroring is advantageous for pre-bending of plates and PSI workflows in moderate to large defects and may reduce intraoperative adjustments while anatomy guided or hybrid approaches may be beneficial in small resections and in cases with condylar involvement [25,27].

### 4.5. Clinical Applicability and Workflow Integration

Although this AI workflow is a combination of all state-of-the-art components like nnU-Net and PCA–ICP, the mirroring process employed has substantial improvements and is superior. It incorporates radiodensity-weighted ICP refinement that enhances accuracy over purely geometric approaches. The algorithm can also mirror defects with due consideration to the density in CT. Additionally, this work has demonstrated quantitative validation and clinical feasibility across both mandibular and cranial defect models.

The fact that both mandibular and cranial cases could be mirrored with similar accuracy, regardless of defect status, highlights the clinical utility of the proposed workflow. The generated models can serve as the basis for preoperative planning, 3D printing of surgical guides, or the design of patient-specific implants. Importantly, the automation of this process reduces dependency on experienced engineers and decreases planning time, making advanced digital workflows more accessible in diverse clinical settings.

The integrated AI-based pipeline achieved a substantial reduction in manual workload compared to traditional methods. In the previously validated version of this workflow, the total time for segmentation and mirroring was reported to decrease from 45–60 min to 3–5 min per case, equivalent to an 85–90% reduction in operator time [30]. Consistent with these findings, the present study reproduced comparable efficiency gains, demonstrating that automation significantly expedites the preoperative planning process without compromising its accuracy.

The system generates ready-to-use mirrored models in STL format, which can be directly imported into CAD/CAM environments such as Materialise 3-matic, Geomagic Freeform, or Medit Model Builder. This compatibility facilitates seamless transition from virtual planning to the design of patient-specific implants or cutting guides, significantly reducing operator time and inter-user variability.

### 4.6. Limitations and Future Work

The present study was limited by the relatively small test set of 40 cases. While this balanced design (10 defect and 10 intact cases per anatomical region) provides strong internal validity it limits the statistical power of the study and generalizability of the findings. Larger multicenter validations are necessary to further assess robustness across diverse patient populations.

Additionally, bilateral defects remain a critical challenge, as no unaffected reference side is available for mirroring. Future approaches may integrate statistical shape modeling or deformable image registration to overcome this limitation. The absence of contralateral refence data may be overcome by the incorporation of shape priors or population- based templates in future research. Another limitation is that the manual reference planes themselves are subject to observer variability, which may partially influence the measured deviations.

Clinical measures such as reconstruction fit in surgical simulations and time saved compared to manual workflows were not assessed in this study and should be considered for future research.

### 4.7. Implications for Surgical Planning

The integration of AI-based segmentation and algorithmic mirroring into a single pipeline offers a step toward fully automated surgical planning workflows. By demonstrating reliable performance across both intact and defective anatomies, this study provides evidence that such workflows can enhance reproducibility, reduce preoperative effort, and broaden access to personalized reconstruction strategies.

## 5. Conclusions

The present study introduced a fully automated workflow that combines deep learning–based segmentation with algorithmic mirroring of craniofacial structures. Validation against expert-generated references confirmed that the method achieves high segmentation accuracy and reliably estimates symmetry planes with angular deviations of approximately one to two degrees. Importantly, the accuracy was consistent across both intact and defective anatomies of the mandible and skull. By eliminating manual interaction, the workflow reduces operator dependency, improves reproducibility, and shortens preoperative planning time. These findings highlight the clinical applicability of automated digital pipelines in craniofacial reconstruction and support their integration into routine surgical planning. Although the present was limited by a small sample size, future studies with larger multicenter datasets and strategies for handling bilateral defects will further strengthen the clinical translation of this approach.

## Figures and Tables

**Table 1:** Acquisition Parameters and Composition of the CT Dataset.

Parameter	Value
Study period	2010–2022
Number of CT scans	388
Region	Head and neck
Slice thickness	0.5–3.0 mm
Mean slices per scan (±SD)	331.56 ± 207.42
Mean voxel size (mm, ±SD)	0.77 × 0.91 × 1.18 ± 0.61–0.91
Resampling	0.5 mm isotropic (B-spline)
Format	DICOM

**Table 2:** Evaluation metrics applied in this study, including their mathematical definitions and corresponding descriptions.

Metric	Formula	Description
Dice similarity coefficient (DSC)	$DSC=\;\frac{2\left|A\cap B\right|}{\left|A\right|+\left|B\right|}=\frac{2\;TP}{2\;TP+FP+FN}$	The **Dice Similarity Coefficient (DSC)** quantifies the spatial overlap between two volumetric datasets, indicating the extent to which the segmented volumes correspond to each other.
Mean surface distance (MSD)	$MSD=\frac{1}{n_{A}+n_{B}}\left(\sum_{i=1}^{n_{A}}\underset{b\in B}{\min}{\left\|a_{i}-b\right\|}_{2}+\sum_{j=1}^{n_{B}}\underset{a\in A}{\min}{\left\|b_{j}-a\right\|}_{2}\right)$	The **Mean Surface Distance (MSD)** measures the average separation between corresponding surface points of the predicted segmentation and the reference, providing an indicator of the overall geometric agreement.
Hausdorff distance (HD)	$\mathrm{HD}=\max{\{\sup}_{x\in X}d\left(x,Y\right),\sup_{y\in Y}d\left(X,y\right)\}$	The **Hausdorff Distance (HD)** represents the greatest distance between points on the predicted segmentation surface and the corresponding points on the reference surface, reflecting the largest boundary discrepancy and therefore the worst-case alignment error.

## Data Availability

The data presented in this study are available on request from the corresponding author.

## Abbreviations

The following abbreviations are used in this manuscript:

3D	Three-Dimensional
AI	Artificial Intelligence
CAD	Computer-aided Design
CAM	Computer-aided Manufacturing
CT	Computed Tomography
DICOM	Digital Imaging and Communications in Medicine
DSC	Dice Similarity Coefficient
HD	Hausdorff Distance
ICP	Iterative Closest Point
MSD	Mean Surface Distance
PCA	Principal Component Analysis
RMS	Root Mean Square
STL	Standard Tessellation Language
VSP	Virtual Surgical Planning
